# Rumen fermentative metabolomic and blood insights into the effect of yeast culture supplement on growing bulls under heat stress conditions

**DOI:** 10.3389/fmicb.2022.947822

**Published:** 2022-09-06

**Authors:** Xian Zhang, Huan Liang, Lanjiao Xu, Bicheng Zou, Tingzhou Zhang, Fuguang Xue, Mingren Qu

**Affiliations:** ^1^Key Laboratory of Animal Nutrition in Jiangxi Province, Jiangxi Agricultural University, Nanchang, China; ^2^ZheJiang Cofine Biotechnology Company Limited, Haining, China; ^3^Yangxin Yiliyuan Halal Meat Co., Ltd., Binzhou, China

**Keywords:** yeast culture, growing bulls, growth performance, metabolomics, heat stress, antioxidant variables

## Abstract

This study was conducted to investigate the effects of yeast culture supplements on the physiological state and growth performance of growing bulls under heat stress conditions and the underlying mechanism. A total of 14 (6.0 ± 1.0 months old) growing bulls with similar body weight were randomly assigned into the control group (YC_0*g/d*_) and yeast culture supplement group (YC_40*g/d*_). YC_0*g/d*_ contained three replicates, with two bulls in each replicate, which were fed a basal diet. Meanwhile, the YC_40*g/d*_ treatment contained four replicates, with two bulls in each replicate, which were fed a basal diet supplemented with 40 g/day of yeast culture per cattle. Growth performance, nutrient digestibility, rumen fermentable metabolites, serum immunity, serum hormones, and serum antioxidant parameters were measured. Results showed that the average daily gain significantly increased (*P* < 0.05), while the feed-to-gain ratio significantly decreased (*P* < 0.01) after YC supplementation compared with the YC_0*g/d*_. The digestibility of neutral detergent fiber (*P* < 0.05) was higher in YC_40*g/d*_. There were no significant differences in ruminal pH, NH_3_-N, butyrate, or acetate/propionate (*P* > 0.05). Besides, the rumen MCP, acetate, propionate, and total VFA content remarkably increased with the supplement of YC (*P* < 0.05). Yeast culture supplementation increased the concentration of nicotinamide riboside, neuromedin B, peptides, and formyl-5-hydroxykynurenamine. The YC_40*g/d*_ group had a significantly (*P* < 0.05) higher serum triiodothyronine level, serum glutathione peroxidase levels, and total antioxidant capacity while having a lower serum malondialdehyde level than the YC_0*g/d*_ group. In conclusion, the addition of yeast culture in the diet improves the growth performance of growing bulls under heat stress by increasing nutrient digestibility, rumen fermentation function, antioxidant capacity, and rumen metabolites.

## Introduction

Heat stress is one of the most stressful events for livestock, which might trigger detrimental consequences for animal health, productivity, and product quality (Gonzalez-Rivas et al., 2020). In terms of ruminants, heat generation was mainly attributed to the high temperature and high humidity combined with the feed fermentation in the rumen, which contributed to the increase in core temperature. Furthermore, long exposure time in the high temperature and humidity environment endangered the physiological ability to regulate temperature, which would be accompanied by a series of changes in physiological functions (Kadzere et al., 2002). Heat stress increases respiration and mortality, reduces fertility, modifies animal behavior, and suppresses the immune and endocrine systems (Thornton et al., 2022). Also, heat stress reduces total volatile fatty acid (VFA) production and leads to changes in rumen pH, and the passage rate and retention time of chyme are also affected by elevated ambient temperature, thereby affecting digestibility (Yadav et al., 2013). Although most relative studies focused on lactating cows, finishing cattle, or calves, correlated studies on growth periods had been rarely reported.

The growth period is critical for the production and economic performance of beef cattle, which requires not only the assurance of adequate necessary nutrients but also the improvement of the immunity and antioxidant function for healthy growth (Galyean et al., 1999). In Euramerican countries, this stage of lifecycle was received continuously grazing, supplemental feed, minerals, and vitamins to guarantee nutritional needs are met for optimal growth, which means less stress and better animal welfare. Nevertheless, in the southern provinces of China, the lack of natural pasture determines that intensive farming systems have to be selected for beef cattle production and may thereafter induce more challenges of stress, especially in summer with high temperatures and high humidity. Therefore, nutrition-dependent regulations were necessary to decrease the negative implication.

Yeast culture (YC) is a product obtained by active yeast through the liquid and solid fermentation, concentrated and dried substances (containing small peptides, amino acids, vitamins, various enzymes, and nucleic acids), extracellular metabolites such as malic acid, and the various nutrients contained in the culture medium after fermentation, the composition of which is mainly determined by the fermentation strain, the medium ratio, and fermentation process conditions. A considerable amount of research has indicated that yeast culture can increase fiber digestibility by optimizing rumen fermentation, relieving stress, improve production performance and animal welfare (Ghazanfar et al., 2019; Perdomo et al., 2020). In Shen’s study, feeding YC performed better or at least equal to antibiotics currently used in beef cattle rations and could be a natural alternative for beef cattle production (Shen et al., 2018).

Metabolomics has provided a useful method to characterize the metabolism of rumen fluid in dairy cows and beef cattle, which can be used to understand the underlying physiological mechanisms that contribute to differences in feed efficiency (Ametaj et al., 2010; Saleem et al., 2012; Artegoitia et al., 2017; Ogunade I. et al., 2019; Clemmons et al., 2020). Although untargeted metabolomics can provide rich information, no research has applied these techniques to analyze the impact of rumen fluid metabolome by YC yet. Therefore, in this study, the objectives were to evaluate the effects of supplemental dietary YC on growth performance and nutrient digestibility in heat-stressed growing bulls. We hypothesized that dietary YC would improve blood parameters, rumen fermentation characteristics, and rumen fluid metabolomics.

## Materials and methods

### Ethical statement

This trial was conducted following the Chinese guidelines for animal welfare and approved by the Committee for the Care and Use of Experimental Animals at Jiangxi Agricultural University (Ethics Approval Code: JXAULL-2021004).

### Animal treatments and experimental design

This research was conducted on the beef cattle research and teaching farm, specifically Yufeng farming in Jiangxi province, China (28.25°N, 115.22°E). The highest temperature in summer in the region often exceeds 38°C.

A total of 14 *Jinjiang* bulls (6 ± 1 months old) with initial body weight (IBW) of 107.5 ± 10.6 kg were randomly allocated into two experimental groups for a 7-day adjustment period followed by a 62-day-long experimental period (69 feeding days in total) feeding process. The YC_0*g/d*_ and YC_40*g/d*_ contained six and eight bulls, respectively. All the bulls were fed in seven pens, with two bulls per pen. The two experimental groups received a YC supplement at 0 or 40 g/day, respectively. YC was acquired from Zhejiang Cofine Biotech. Co., Ltd. (Haining, Zhejiang, China). The fermentation strain is *Saccharomyces cerevisiae*. All bulls were regularly provided rations twice a day at 09:00 and 16:00. Bulls had free access to water during the experiment.

The temperature and humidity were recorded by a Temperature & Humidity Recorder (Renke COS-04, Renke Control Technology Co., Shangdong, China) at 9:00 and 16:00 h each day, and the temperature and humidity index (THI) was calculated using the following formula:


THI=(1.8×T+32)-[(0.55-0.55×RH)×(1.8⁢T-26)],

where *T* is temperature (°C) and RH is relative humidity percentage (%) (Domínguez-Mancera et al., 2017).

**FIGURE 1:**
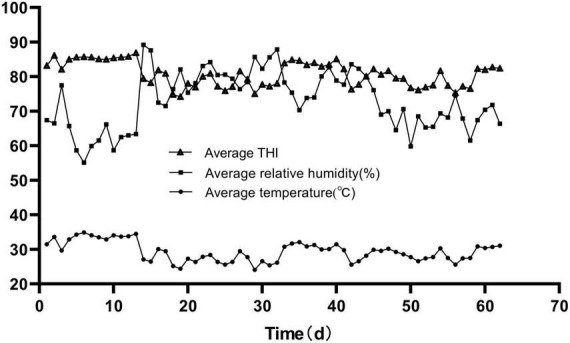
The average temperature, average relative humidity, and THI during the trial period.

As shown in Figure 1, the average daily THI values during the experimental period were higher than 74 for the whole trial. THI values were interpreted as follows: ≤ 74, comfort; 75–78, alert; 79–83, dangerous; and ≥84, emergency (Domínguez-Mancera et al., 2017). The growth bulls were suffering from heat stress throughout the whole trial period.

The basal diet (Table 1) was designed to support the nutrient needs of protein, energy, vitamins, and minerals, which is consistent with the feeding standards of beef cattle in China, NY/T 815-2004 (Ministry of Agriculture of People’s Republic of China 2004). The ratio of concentrate:roughage was 40:60 DM (dry matter). The concentrate consisted of corn, soybean meal, NaHCO_3_, and premix. The roughage included straw and Baijiu fermented grains.

**TABLE 1:** Ingredients and nutrient levels of the experimental diets (dry matter basis).

Ingredients, %	Content (%)	Nutrient levels	Content (%)
Corn	31.00	Dry matter	72.35
Soybean meal	6.00	Crude protein	13.35
straw	34.00	Ash	8.40
Baijiu fermented grains	26.00	Neutral detergent fiber	43.15
NaHCO_3_	0.80	Acid detergent fiber	21.91
NaCl	0.40	Ca	0.89
Premix*^a^*	1.80	P	0.61

^*a*^One kilogram of premix provided the following: vitamin A, 150,000 IU; vitamin D3, 20,000 IU; vitamin E, 3,000 IU; Fe 3, 750 mg; Mn 3, 200 mg; Zn, 2,000 mg; Cu, 600 mg; I, 20 mg; Se, 10 mg; Co, 10 mg; Ca, 130 g, P, 30 g.

Dry matter intake (DMI) was recorded daily for each pen, and the BW of each bull was measured at the beginning and end of the trail, with an empty stomach in the morning. The recorded date was used to calculate the average daily gain (ADG).

### Determination of apparent nutrient digestibility

Representative feed and fecal samples were collected during the last 3 days in two stages (days 28–30 and 60–62 of the experimental period) after the morning feeding, and results are displayed as the average of the total 3 days. Fecal samples were collected by the rectal sampling method. All feces were evenly mixed, and 10% H_2_SO_4_ was added to fix nitrogen, then stored at −20°C. Feed and fecal samples were dried in a forced air oven at 65°C for 72 h and then ground by the Wiley mill through a 1-mm screen sieve.

Dry matter (DM), crude protein (CP), crude fat (CF), ash, and ether extract (EE) of the feed and fecal samples were determined according to the Association of Analytical Communities (AOAC) using the methods 967.03, 984.13, 920.39, 924.05, and 920.39, respectively. Neutral detergent fiber (NDF) and acid detergent fiber (ADF) were determined using an Ankom A200i Fiber analyzer (ANKOM Technology Co., New York, NY, United States) according to Van Soest (Van Keulen and Young, 1977). The content of hydrochloric acid insoluble ash (AIA) was determined according to Block (Block et al., 1981), and AIA was used as a digestibility marker. The formula was as follows:


AD(%)=[(a/c-b/d)/(a/c)]×100

*AD* represents the apparent digestibility of nutrient content, *a* represents the nutrient content of the feed, *b* represents the nutrient content of feces, *c* represents the AIA content of the feed, and *d* represents the AIA content of feces.

### Determination of rumen fermentation characteristics

Rumen fluid samples of 12 bulls (six bulls in each group, similar body weight) were collected on the last day through esophageal tubing in the morning before feeding. The first 150 ml of rumen fluid sample was discarded to minimize saliva contamination. The rumen fluid pH was measured immediately using a portable pH meter [Testo 206-pH1, Testo Instruments International (Shanghai) CO., Shanghai, China]. At the same time, 5 ml of rumen fluid was preserved at −80°C for further test and other samples were processed to analyze VFA, microbial protein (MCP), and ammonia-N (NH_3_-N). The ruminal MCP concentration was detected with a bicinchoninic acid protein quantification kit (Yeasen Biotechnology), and the concentration of NH_3_-N was determined using alkaline sodium hypochlorite-phenol spectrophotometry. The concentrations of VFA were determined by a gas chromatograph (GC-2014, Shimadzu, Tokyo, Japan) equipped with a capillary column (Stabilwax, Restek, Bellefonte, PA, United States). Notably, 1.00 ml of rumen supernatant was added to 0.20 ml of 25% (w/v) metaphosphoric acid and centrifuged at 10,000 r/min for 15 min. Then, the supernatant was used for the VFA test.

### Metabolomics analysis of rumen fluid

For metabolomics, 12 rumen fluids from YC_0*g/d*_ and YC_40*g/d*_ were used. Non-targeted profiling of metabolites in rumen fluid was obtained according to the instructions of Majorbio Bio-Pharm Technology Co., Ltd. (Shanghai, China). A fluid sample of 100 μl was accurately weighted, and the metabolites were extracted using a 400 μl of cold methanol:water (4:1, v/v) solution. The mixture was treated by the high throughput tissue crusher Wonbio-96c at 50 Hz for 6 min, then followed by vortex for 30 s and ultrasound at 40 kHz for 30 min at 5*^o^*C. The samples were placed at −20*^o^*C for 30 min to precipitate proteins. After centrifugation at 13,000 g at 4*^o^*C for 15 min, the supernatant was taken for LC-MS (Waters ACQUITY Ultra-Performance Liquid Chromatography System, Milford, United States) coupled with a triple time-of-flight mass spectrometer (AB SCIEX TripleTOF 5600 System, Framingham, United States). A preheated hyper ACQUITY BEH C18 column (100 mm × 2.1 mm, 1.7 μm; Waters, Milford, United States) was used for chromatographic separation in the positive ion mode (ESI+) and negative ion modes (ESI-). The column temperature was maintained at 40*^o^*C. The sample injection volume was 2 μl, and the flow rate was set to 0.4 ml/min. The quality control samples consisting of equivalent mixtures of all rumen fluid samples were analyzed regularly to monitor the stability of the analysis.

### Blood sampling and analysis

Blood samples were collected from the jugular vein into evacuated non-anticoagulative tubes before morning feeding on days 30 and 62. The blood samples were centrifuged at 3,000 g for 10 min to obtain serum samples and then stored at −20°C until required for further analysis.

The concentrations of serum immunoglobulin A (IgA), immunoglobulin B (IgG), immunoglobulin M (IgM), growth hormone (GH), interleukin-4 (IL-4), interleukin-6 (IL-6), tumor necrosis factor-α (TNF-α), cortisol (COR), triiodothyronine (T3), and thyroxine (T4) were measured using corresponding commercial kits (Beijing Sino-UK Institute of Biotechnology Technology, Beijing, China) and a microplate reader (DR-200BS, Wuxi DR-200BS, Wuxi, China).

The concentrations of serum malondialdehyde (MDA), the activities of superoxide dismutase (SOD), glutathione peroxidase (GSH-Px), and total antioxidant capacity (T-AOC) were measured with commercial kits (Nanjing Jiancheng Bioengineering Institute) and an Epoch 17121332 enzyme marker (Bio Tek, Vermont, United States).

### Data analysis

A normal distribution test on growth performances and rumen fermentation analysis was first conducted using SPSS (Statistical Product Service Solutions) 23.0 software “proc univariate data = test normal” followed by a Student *t*-test (two-tail test). ANOVA repeated measures were performed to analyze digestibility and serum parameters at different time points (30 or 62 days). The results are shown as the mean and standard error mean (SME). *P*-value < 0.05 was considered to be significant.

The metabolomics data were subjected to partial least squares discriminant analysis (PCA), orthogonal partial least squares discriminate analysis (OPLS-DA), and differential metabolite screening on Majorbio Cloud Platform,^1^ with a false discovery rate (FDR) < 0.05 and variable weight importance ranking (VIP) > 1 as the screening criteria.

## Result

### Growth and feed efficiency

As shown in Table 2, there were no significant differences in ADFI (average daily feed intake), IBW (initial body weight), and FBW (final body weight) among groups (*P* > 0.05). However, the ADG of bulls was higher in the YC_40*g/d*_ group than in the YC_0*g/d*_ group (*P* < 0.05). The YC_40*g/d*_ group had a lower F:G value than the YC_0*g/d*_ group (*P* < 0.01).

**TABLE 2:** Effect of feeding YC on growth and feed efficiency of growing bulls.

Items	YC_0*g/d*_	YC_40*g/d*_	*P*-value
ADFI (kg/d)	3.58 ± 0.05	3.70 ± 0.06	0.241
IBW (kg)	112.8 ± 2.99	116.6 ± 3.86	0.368
FBW (kg)	151.9 ± 3.18	160.9 ± 3.73	0.141
ADG (kg/d)	0.566 ± 0.018^b^	0.643 ± 0.019^a^	0.035
F:G	6.33 ± 0.10^a^	5.76 ± 0.07^b^	0.006

The results are presented as the mean value ± SEM. Mean values with different letters are significantly different (*P* < 0.05).

SEM, standard error of the mean; YC, yeast culture; ADFI, average daily feed intake; IBW, initial body weight; FBW, final body weight; ADG, average daily gain; F:G, feed to gain ratio.

### Nutrient digestibility

The results of nutrient digestibility are presented in Table 3. There was no significant interaction between YC addition and time on the digestibility of feed. Supplementation with YC significantly increased the digestibility of NDF (*P* < 0.05). Except for ADF, the time of measurement had no effect on the group differences between treatments.

**TABLE 3:** Effect of feeding YC on nutrient digestibility of growing bulls on days 30 and 62.

Item	Time (d)	YC_0*g/d*_	YC_40*g/d*_	*P*-value		
				Time	YC	Time × YC
DM %	30	70.37 ± 1.12	70.83 ± 1.46	0.342	0.558	0.776
	62	70.63 ± 1.04	71.47 ± 0.85			
NDF %	30	59.21 ± 1.70	63.68 ± 2.84	0.081	0.037	0.644
	62	57.23 ± 2.17	61.24 ± 2.14			
ADF %	30	52.33 ± 0.87	52.83 ± 1.26	0.003	0.366	0.201
	62	56.36 ± 1.64	54.67 ± 1.67			
CP %	30	64.80 ± 2.11	63.13 ± 4.83	0.101	0.189	0.514
	62	59.96 ± 2.25	63.73 ± 0.52			
CF %	30	79.51 ± 3.14	82.40 ± 2.87	0.169	0.558	0.404
	62	77.87 ± 2.66	76.65 ± 3.77			

The results are presented as the mean values ± SEM.

SEM, standard error of mean; YC, yeast culture; DM, dry matter; NDF, neutral detergent fiber; ADF, acid detergent fiber; CP, crude protein; CF, crude fat.

### Rumen fermentation

As shown in Table 4, the levels of rumen MCP, acetate, propionate, and total VFA were increased significantly (*P* < 0.05) in the YC_40*g/d*_ group when compared with the YC_0*g/d*_ group. There were no significant differences in ruminal pH, NH_3_-N, butyrate, or acetate/propionate (*P* > 0.05).

**TABLE 4:** Effect of feeding YC on rumen fermentation of growing bulls.

Item	YC_0*g/d*_	YC_40*g/d*_	*P*-value
Ruminal pH	6.28 ± 0.03	6.30 ± 0.02	0.658
MCP (mg/dL)	69.09 ± 2.11^a^	76.27 ± 2.09^b^	0.036
NH_3_-N (mmol/dL)	11.09 ± 1.20	12.94 ± 1.07	0.274
Acetate (mmol/L)	32.01 ± 1.19	38.50 ± 1.70	0.011
Propionate (mmol/L)	8.49 ± 0.68^a^	11.50 ± 0.74^b^	0.013
Butyrate (mmol/L)	4.45 ± 0.37	5.43 ± 0.40	0.103
Acetate/propionate	3.84 ± 0.19	3.42 ± 0.24	0.197
Total VFA (mmol/L)	46.73 ± 2.25^a^	57.98 ± 1.92^b^	0.004

The results are presented as the mean values ± SEM. Mean values with different letters are significantly different (*P* < 0.05)

SEM, standard error of the mean; YC, yeast culture; MCP, microbial crude protein; NH_3_-N, ammonia nitrogen; VFA, volatile fatty acid.

### Rumen fluid metabolomics

**FIGURE 2:**
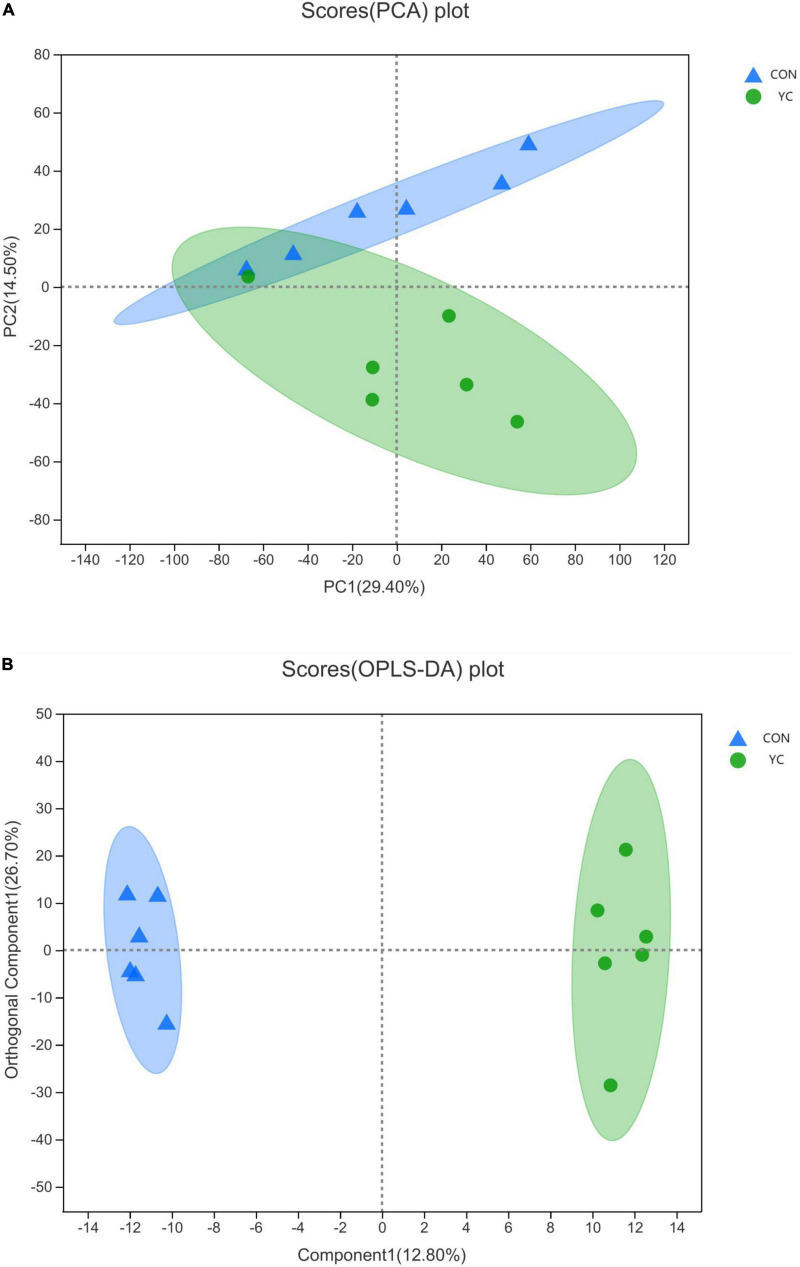
**(A)** A PCA of ruminal metabolites from bulls (*n* = 6) fed a control diet (control) and bulls fed 40 g/day of YC; **(B)** an OPLS-DA of ruminal metabolites from bulls (*n* = 6) fed a control diet (control) and bulls fed 40 g/day of YC.

Rumen fluid metabolites from YC_0*g/d*_ and YC_40*g/d*_ were analyzed by LC-MS. A total of 777 differential peaks were selected, including 387 peaks in ESI+ and 390 peaks in ESI-. The PCA showed a primary unsupervised separation between the two groups (Figure 2A). To better distinguish the differences between the groups and improve effectiveness, an OPLS-DA score plot was performed to supervise the multivariate analysis (Figure 2B). The two groups can be clearly separated.

As shown in Table 5, there were 29 differential metabolites between the YC_0*g/d*_ and YC_40*g/d*_ groups with a variable importance projection (VIP) value > 1.0 and *P* < 0.05.

**TABLE 5:** Different metabolite contents in the rumen of bulls fed a control diet (CON) vs. bulls fed 40 g/day of YC.

Classification	Metabolites	FC (CON/YC)	VIP	*P*-value
Amino acids and peptides metabolism	ACRL Toxin II	0.983	1.258	0.012
	Atemoyacin B	0.987	1.212	0.004
	Neuromedin B	0.744	4.889	0.000
	Phenylalanyl-Lysine	0.905	3.239	0.000
	Glycyl-prolyl-hydroxyproline	0.934	2.375	0.017
	2-{[hydroxy(1H-indol-3-yl)methylidene]amino}acetic acid	0.980	1.400	0.005
	(S)-2,3,4,5-Tetrahydropiperidine-2-carboxylate	0.979	1.332	0.023
	Gamma-Glutamyl-S-methylcysteine sulfoxide	1.014	1.083	0.028
carbohydrates metabolism	Ferulic acid 4-sulfate	1.247	4.044	0.006
	Nicotinamide riboside	0.936	2.226	0.044
	Formyl-5-hydroxykynurenamine	0.912	2.644	0.014
Lipometabolic metabolites	Tributyrin	0.799	4.237	0.001
	3-Indolebutyric acid	0.900	2.898	0.005
	6-Hydroxykynurenic acid	1.105	2.903	0.014
	Xanthurenic acid	1.105	2.827	0.016
	P-Salicylic acid	1.064	2.733	0.000
	Hypogeic acid	0.963	1.829	0.019
	Eriojaposide B	1.067	2.272	0.028
	Gamma-Tocotrienol	0.945	2.400	0.004
	(24R)-Ergost-4-ene-3,6-dione	0.965	1.839	0.017
	N-[(4E,8E)-1,3-dihydroxyoctadeca-4,8-dien-2-yl]hexadecanamide	1.039	1.814	0.013
	11-Hydroxyeicosatetraenoate glyceryl ester	0.988	1.078	0.021
	(6beta,7alpha,12beta,13beta)-7-Hydroxy-11,16-dioxo-8,14-apianadien-22,6-olide	1.058	2.075	0.026
	Isofucosterol glucoside	1.032	1.685	0.026
	(20R)-Ginsenoside Rh2	1.034	1.641	0.034
	4,8 dimethylnonanoyl carnitine	0.976	1.469	0.020
	2beta,9xi-Dihydroxy-8-oxo-1(10),4,11(13)-germacratrien-12,6alpha-olide	0.973	1.585	0.025
	27-Norcholestanehexol	0.988	1.119	0.004
	Isoamberboin	1.019	1.205	0.020

All different metabolites listed here are those VIP > 1 and *P*-value < 0.05.

FC, fold change.

FC values < 1 mean that metabolites are greater in YC and FC values > 1 mean that the metabolite is lower in YC.

**FIGURE 3:**
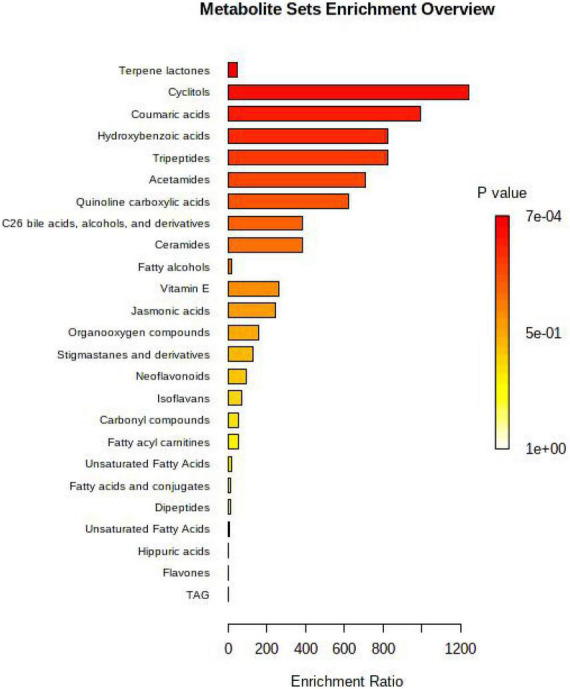
Analysis of the KEGG pathway enriched by differential metabolites in the rumen.

The detected differential metabolites were screened, and the metabolites that could be enriched in the metabolic pathways in the KEGG database were selected for visual analysis of their metabolic pathways. Figure 3 shows the metabolic pathways enriched in the rumen. Based on the pathways of the YC_40*g/d*_ group vs. control group were enriched, which included terpene lactones, cyclitols, coumaric acids, tripeptides, acetamide, quinoline carboxylic acid, C26 bile acids, alcohol, and derivatives, ceramides, fatty alcohols, vitamin E.

### Blood immunological function, hormone, and antioxidant parameters

As presented in Table 6, there was no significant interaction between YC addition and time on blood immunological function, hormone, or antioxidant parameters. Supplementation with YC significantly decreased serum MDA (*P* < 0.05) and increased the concentration of serum T4 (*P* < 0.05), GSH (*P* < 0.01), and T-AOC (*P* < 0.05). Except for MDA, the time of measurement had no effect on the group differences between treatments.

**TABLE 6:** Effect of feeding YC on the immunological function, anti-stress, and antioxidant parameters in the serum on days 30 and 62.

Item	Time (d)	YC_0*g/d*_	YC_40*g/d*_	*P*-value		
				Time	YC	Time × YC
IgA, g/L	30	2.00 ± 0.36	1.91 ± 0.30	0.392	0.096	0.582
	62	2.32 ± 0.42	1.98 ± 0.16			
IgG, g/L	30	15.21 ± 2.65	15.29 ± 3.70	0.387	0.986	0.974
	62	16.53 ± 2.92	16.51 ± 1.94			
IgM, g/L	30	1.39 ± 0.20	1.36 ± 0.21	0.127	0.909	0.857
	62	1.56 ± 0.39	1.57 ± 0.15			
IL-4, pg/ml	30	7.40 ± 4.23	6.46 ± 1.86	0.490	0.778	0.476
	62	7.35 ± 2.19	9.44 ± 6.01			
IL-6, pg/ml	30	122.3 ± 20.3	144.1 ± 29.7	0.064	0.172	0.287
	62	100.4 ± 14.6	99.8 ± 19.8			
TNF-α, pg/ml	30	62.92 ± 10.07	50.18 ± 7.38	0.090	0.227	0.076
	62	50.77 ± 5.23	47.75 ± 5.40			
COR, ng/ml	30	76.08 ± 9.15	75.73 ± 8.56	0.234	0.528	0.735
	62	67.67 ± 20.76	61.13 ± 15.17			
T3, ng/ml	30	0.91 ± 0.14	1.11 ± 0.19	0.136	0.064	0.229
	62	0.89 ± 0.18	0.85 ± 0.13			
T4, ng/ml	30	53.90 ± 4.65	68.74 ± 9.04	0.245	0.015	0.738
	62	52.15 ± 5.41	65.67 ± 5.99			
SOD, U/L	30	17.17 ± 2.8	17.06 ± 2.10	0.799	0.990	0.815
	62	16.99 ± 2.96	17.05 ± 2.50			
MDA, mmol/L	30	4.34 ± 0.27	3.60 ± 0.49	0.005	0.017	0.324
	62	4.77 ± 0.34	4.23 ± 0.09			
GSH, U/L	30	27.86 ± 6.66	41.20 ± 9.85	0.128	0.005	0.935
	62	35.90 ± 5.56	48.50 ± 6.94			
T-AOC, mmol/L	30	2.82 ± 0.25	3.01 ± 0.40	0.297	0.045	0.211
	62	2.45 ± 0.22	3.05 ± 0.31			

The result is presented as the mean values ± SEM.

SEM, standard error of mean; YC, yeast culture; MDA, malondialdehyde; SOD, superoxide dismutase; GSH-Px, glutathione peroxidase; T-AOC, total antioxidant capacity.

## Discussion

This study has been widely used as a practical indicator of the heat stress degree (Habeeb et al., 2018). Livestock Weather Safety Index was developed by Livestock Conservation, Inc. and indicated that THI indexes greater than 74 were considered the occurrence of heat stress (Domínguez-Mancera et al., 2017). In this trial, the growing bulls spent the whole period of 62 days in THI > 74 conditions and a total of 40 days in THI > 79. This suggests that the growing bulls suffered from heat stress throughout the whole trial period.

### Yeast culture improves the growth performance of heat-stressed growing bulls

Heat stress leads to a decrease in feed intake, digestion, and absorption of nutrients, resulting in poor growth performance of cattle (O’Brien et al., 2010; Wang et al., 2020). YC contains mass active yeast cells and compounds produced during fermentation, such as organic acid and VB, which showed positive effects on animals’ growth performance and health. In our study, there was an increasing ADG and a reducing F:G ratio in response to YC supplementation, and these findings were in line with a meta-analysis by Wagner (Wagner et al., 2016) and L. Armato’s study (Armato et al., 2016). Both of them showed that feeding beef cattle with YC enhanced the production performance, which included improved ADG, feed conversion ratio, rumen fermentation, and carcass quality.

Although feed intake was not affected by YC, increased NDF digestibility provides more nutrients to the growing bulls and further significantly improves growth performance. YC increases the level of nutrient metabolism (especially the carbohydrates) in the rumen, perhaps the most important reason for the improved production performance.

### Effects of yeast culture supplementation on carbohydrate metabolism

The metabolic processes involving carbohydrates, fat, protein, and vitamins can help explain the difference in feed efficiency. However, the role of most of the differential rumen metabolites detected in this trial has not been described, which was similar to Ogunade’s study on live yeast (Ogunade I. M. et al., 2019).

Rumen pH was not influenced in this study, possibly because of the non-acidotic diet fed, which concurred with Gattass’ study (Gattass et al., 2008). Ruminal pH directly reflects the health of rumen fermentation function. The study found adding yeast increased the activity of lactate-utilizing bacteria and reduced the proliferation of lactate-producing bacteria, thus preventing lactic acid accumulation and increasing rumen pH (Gattass et al., 2008).

In this trial, NDF digestibility and VFA levels were significantly improved. The ability of yeast to stimulate the growth of fiber-digesting bacteria has been confirmed. Evidence suggests that the yeast can provide nutrients that stimulate the growth of cellulolytic bacteria such as *Ruminococcus albus*, *Fibrobacter succinogenes*, and *Ruminococcus flavefaciens* (Chaucheyras-Durand et al., 2008). Changes in the rumen microbiota, especially the abundance of fiber-digesting bacteria, lead to changes in the type and proportion of individual VFA produced in the rumen. The contents of acetate, propionate, and total VFA were significantly increased in this experiment. As the main source of glucose supplement for ruminants and a major substrate for glucose genesis, propionate will be rapidly absorbed by the rumen papilla and used for energy (Dijkstra et al., 2012). Butyrate is a direct energy donor for rumen epithelial cells and rumen papilla development (Laarman et al., 2012). Some experiments (Thrune et al., 2009; Zhu et al., 2017) found that the concentration and ratio of butyrate in the rumen increased after adding YC.

Nicotinamide riboside (NR) is a precursor of nicotinamide adenine dinucleotide (NAD) and a major NAD+ precursor vitamin in cow milk (Trammell et al., 2016), and represents a source of niacin (vitamin B3). Niacin is particularly important for ruminants, participating in the metabolism of carbohydrates, proteins, and fats, and can improve the synthesis capacity of rumen microbial protein and increase the content of rumen propionic acid (Trammell et al., 2016). Meanwhile, niacin synthesis in anaerobic microbial systems uses 3- and 4-carbon precursors such as propionate, glycerol, malic acid, succinic acid, or fumaric acid. In this trial, the YC_40*g/d*_ group had increased propionate levels, which may explain the changes in VFA. Studies have found that supplementation of yeast cultures in heat-stressed dairy cows could increase plasma nicotinic acid concentrations (Salvati et al., 2015; Dias et al., 2018). Therefore, it is reasonable to suspect that yeast can increase the production of niacin and its related products in the rumen.

### Effects of yeast culture supplementation on nitrogen metabolism

Supplementation with 40 g/day YC in the diet significantly increased the nitrogen-metabolic related contents of neuromedin B, phenylalanyl-lysine, and glycyl-prolyl-hydroxyproline. Neuromedin B regulates exocrine and endocrine secretion, blood glucose, body temperature, energy homeostasis, and cell growth (Salvati et al., 2015; Dias et al., 2018). However, its effects on rumen function in ruminants have not been reported. Phenylalanyl-lysine and glycyl-prolyl-hydroxyproline belong to the dipeptide and tripeptide, respectively. Peptides are potential nutrients for the growth of ruminal microorganisms but are also liable to be degraded to ammonia and lost from the rumen. The growth of mixed rumen bacteria is primarily driven by carbohydrate fermentation but is greatly stimulated by peptides that act as multipliers for microbial growth (Argyle and Baldwin, 1989), and this may help to explain the elevated MCP levels in rumen.

Formyl-5-hydroxykynurenamine is a metabolite of 5-hydroxytryptamine (5-HT). 5-HT is produced by tryptophan through the two-stage enzymatic reaction of tryptophan hydroxylase and aromatic amino acid decarboxylase and is involved in the tryptophan metabolism pathway (Bai et al., 2017). The supplement of YC could improve the metabolic utilization of 5-HT and the latter is involved in the hypothalamic-pituitary-adrenal axis (HPA) to help regulate stress (Dinan, 1996). This might help enhance the body’s stress-resistant capacity, improve body health, and further promote gastrointestinal digestibility.

### Yeast culture improves serum antioxidant capacity

We found the addition of yeast culture in the diet can effectively improve the serum antioxidant indicators of growing bulls, which was consistent with the result of the previous study (Broadway et al., 2015). Under the condition of heat stress, the metabolic process of the animal body is disordered, the production of oxygen free radicals increases, and the antioxidant activity decreases so that the body cannot effectively use antioxidant enzymes to remove free radicals in a timely manner, resulting in an accumulation of free radicals and lipid peroxidation in the body reaction (Belhadj et al., 2016). The body needs to consume a large amount of antioxidant enzymes to scavenge excessive free radicals, resulting in a decrease in the activity of superoxidase dismutase, glutathione peroxidase, and total antioxidant capacity and an increase in the production of malondialdehyde (Sordillo and Aitken, 2009). Pancini (Pancini et al., 2020) considered that the yeast products would be of greater benefit to feedlot cattle experiencing stressful conditions. In this trial, coumaric acid (CA) is a phenolic acid of the hydroxycinnamic acid family, and it has many biological functions such as antioxidant, anti-inflammatory, antidiabetic, anti-ulcer, antiplatelet, and anticancer activities. Gamma-tocotrienol as a vitamin E isoform, a natural dietary antioxidant (Pathak et al., 2016), was significantly increased after YC supplementation compared with control. This may help to increase the body’s antioxidant capacity. In this study, time treatment significantly increased the serum MDA, which means that with the prolongation of heat stress, the body’s peroxidation response is strengthened.

Yeast cell walls consist of β-glucans, and mannan oligosaccharides (MOS) appear to possess the ability to bind the receptors of defense cells of the gut, which activates animal immune defenses such as phagocytosis and decreases morbidity (Broadway et al., 2015). Burdick found that supplementation of yeast products may improve energy availability during an immune challenge (Burdick et al., 2020). However, in this trial, the addition of yeast culture under a heat stress environment did not affect the immune function of the body, which was similar to a previous study (Ma et al., 2015).

As the major thyroid hormones, T3 and T4 are key regulators of energy metabolism in the body. To better adapt to the hot environment, the thyroid activity in animals will be relatively reduced, thereby reducing the release of thyroid hormones (Pereira et al., 2008; Sejian et al., 2010). In this study, compared with the control group, the content of serum T4 increased with the diet supplemented with YC; however, T3 and COR showed no difference. This may be related to the physical differences between different animals.

## Conclusion

In conclusion, dietary supplementation with 40 g/day YC improved feed efficiency and increased antioxidant status and ruminal VFA that enhanced the production performance of growing bulls under heat stress. After adding YC, related metabolites such as NR and small peptides in the rumen increased. This partly explains the variation in rumen fermentation parameters. This study enhances our understanding of the effects of YC in the rumen, and more research is needed in the future.

## Data availability statement

The original contributions presented in this study are included in the article/Supplementary material, further inquiries can be directed to the corresponding authors.

## Ethics statement

The animal study was reviewed and approved by the Committee for the Care and Use of Experimental Animals at Jiangxi Agricultural University. Written informed consent was obtained from the owners for the participation of their animals in this study.

## Author contributions

MQ designed the overall study. XZ, FX, HL, LX, BZ, and TZ performed the experiments. XZ and FX wrote the manuscript. All authors contributed to the article and approved the submitted version.

## Footnotes

1
https://cloud.majorbio.com
